# Positional differences in the micro- and ultra-structural variations of ray parenchyma cells during the transformation from sapwood to heartwood

**DOI:** 10.3389/fpls.2024.1431818

**Published:** 2024-09-03

**Authors:** Lijuan Yin, Lingyu Ma, Xiaomei Jiang, Yonggang Zhang, Yupei Wei, Yuan Cao, Lihong Yao, Juan Guo

**Affiliations:** ^1^ Research Institute of Wood Industry, Chinese Academy of Forestry, Beijing, China; ^2^ College of Material Science and Art Design, Inner Mongolia Agricultural University, Hohhot, China; ^3^ State Key Laboratory of Tree Genetics and Breeding, Chinese Academy of Forestry, Beijing, China

**Keywords:** ray parenchyma cell, contact cell, isolation cell, ultrastructure, heartwood, sapwood, transmission electron microscope

## Abstract

Ray parenchyma cells are involved in the initiation of heartwood formation. The position within a ray influences the timing of ray parenchyma cell differentiation and function; however, there is little information concerning the positional influence on the cellular changes of ray parenchyma cells from sapwood and heartwood. In this study, radial variations in morphology, size, and ultrastructure of ray parenchyma cells were studied by combined transmission electron microscopy and optical microscopy. Results showed that cellular traits of ray parenchyma cells in *Populus tomentosa* were all affected by both radial position in the secondary xylem and position within a ray. Specifically, radial variations in cellular traits were more evident in isolation cells, which were not adjacent to vessel elements. Both cell length and cell width/length ratio of isolation cells were bigger than contact cells, which contacted adjacent vessel elements via pits. Moreover, the secondary wall thickening and lignification of contact cells developed in the current-year xylem, much earlier than isolation cells. Secondary walls in contact cells were in a polylamellate structure with a protective layer on the inner side. No alteration in the ultrastructure of contact cells occurred in the sapwood-heartwood transition zone, except that most contact cells died. By contrast, in the transition zone, isolation cells still lived. A thin secondary wall began to deposit on the thick primary wall of isolation cells, with two isotropic layers on the inner side of the primary wall and secondary wall respectively being characteristic. Meanwhile, starch grains in isolation cells were depleted, and dark polyphenolic droplets lost their spherical shape and flowed together. Furthermore, the intercellular spaces of isolation cells became densified in the transition zone. Overall, cellular changes suggested that the positional information of ray parenchyma cells appeared to be an important factor in the transformation from sapwood to heartwood. Unlike contact cells, isolation cells were more elongated, specialized in radial transport, had a delayed formation of secondary walls, and were involved in the synthesis of heartwood substances. Our result promotes the elucidation of the involvement of xylem rays in heartwood formation.

## Introduction

Xylem cells within a tree undergo several unique and fascinating biological processes on the way from the cambium to the heartwood (Spicer and Holbrook, 2007; Cuny et al., 2018; Zhang et al., 2022; German et al., 2023). One may distinguish two main steps resulting from the ripening of inward-directed cambial derivatives (Kampe and Magel, 2013; Tung et al., 2023; Fangel et al., 2024). The first one is the formation of the wood cell, especially regarding the vascular elements, which have always been of interest (Zhang et al., 2022; Sajjad et al., 2023; Xue et al., 2023). The second step is heartwood formation, the final step in the life cycle of living xylem cells (Spicer, 2005; Ma et al., 2023). Despite the ecological and economic significance of heartwood (Taylor et al., 2002; Viotti et al., 2021; Ren et al., 2023), the regulation of heartwood formation has not yet been completely clarified. Several essentially different hypotheses have been put forward to interpret the mechanism of heartwood formation, for instance, the death of parenchyma cells, gas accumulation, desiccation, and the involvement of gene expression and enzymatic activities (Rudman, 1966; Spicer, 2005; Liu et al., 2022). Among them, it is thought that a complicated system of combined reactions within the parenchyma cells would greatly contribute to the heartwood formation.

Ray parenchyma cells, extending radially in the stem of trees, are involved in the initiation of heartwood formation (Bamber, 1976; Nagai and Utsumi, 2012; Lim et al., 2016). They generally do not have lignified walls within the zone of developing cells, participate in the biosynthesis and accumulation of heartwood substances at the transition zone between sapwood and heartwood (Magel et al., 1994; Zhang et al., 2009; Chen et al., 2018), and commonly die in middle and inner heartwood (Song et al., 2011; Kampe and Magel, 2013). Ray parenchyma cell walls also change gradually towards heartwood (Song et al., 2011; Kampe and Magel, 2013; Zheng et al., 2014, 2016). Therefore, the influence factors and change path related to the transformation of ray parenchyma cell walls from sapwood to heartwood can provide important clues to an understanding of the heartwood formation process.

Ray parenchyma cells present positional differences in terms of their contacts with axial xylem elements; contact cells are ray cells that connect with vessel elements or ray tracheids directly through pits, and isolation cells are ray cells that do not make any direct connections with vessel elements or ray tracheids through pits (Murakami et al., 1999; Nakaba et al., 2006). They differ in the timing of cell differentiation, death, and function (Vogelmann et al., 1985; Nakaba et al., 2006, 2012; Tulik et al., 2019). The formation of the secondary wall takes place earlier in contact cells (Murakami et al., 1999; Yin et al., 2022), tyloses develop from contact cells and not from isolation cells (Micco et al., 2016), and isolation cells might be more specialized for radial transportation than contact cells (Sauter and Kloth, 1986). Furthermore, structural features also differ between contact cells and isolation cells. Isolation cells have a longer and narrower cell morphology with much thicker cell walls than contact cells, moreover, the pits of isolation cells are smaller and more bordered, with a greater pit density, especially on end walls (Yin et al., 2022). It seems possible that these two types of ray parenchyma cells might influence heartwood formation via different processes. However, little attention has been paid to the influence of positional differences in ray parenchyma cells in relation to heartwood formation.

Our main objective in this study was to test the hypothesis that positional differences in ray parenchyma cells might influence the ultrastructural variation during the transformation from sapwood to heartwood. By using mature 48-year-old *Populus tomentosa* trees, ray parenchyma cells within a ray and proximity to neighboring short-lived vessel elements were studied comparatively. Micro- and ultra-structural variations of ray parenchyma cells during the transformation from sapwood to heartwood were studied, including the morphology, quantitative anatomic analysis, histochemical analysis, and cell wall ultrastructure of ray parenchyma cells. We then discuss the influence of positional differences underlying these changes. The results will elucidate the involvement of ray parenchyma cells in heartwood formation.

## Materials and methods

### Plant material

The specimens used in this study were the radial wood cores of 48-year-old *Populus tomentosa* trees growing at the Chinese Academy of Forestry (116°24′N, 40°00′E). All samples were obtained at breast height in October, because the processes of heartwood formation are initiated in late summer, with the highest activity in autumn and cessation during dormancy (Kampe and Magel, 2013). Samples were immediately placed in a mixture solution of formalin, acetic acid, and ethanol (FAA), vacuum-pumped for 1 hour, and finally stored in the refrigerator at 4°C.

### Ray parenchyma cells were chosen at five radial positions in the secondary xylem

To characterize ray parenchyma cells during a gradual transformation from sapwood and heartwood, five radial positions in the xylem parts of the wood radial cores were selected for this study: (*R1*) the current-year xylem: the 1^st^ growth ring; (*R2*) middle sapwood: the growth ring midway between cambium and heartwood, the 15^th^ growth ring; (*R3*) inner sapwood: the 20^th^ growth ring; (*R4*) transition zone: the 23^rd^ growth ring; (*R5*) middle heartwood: the growth ring midway between transition zone and heartwood, the 33^rd^ growth ring. The timing of differentiation and death of ray parenchyma cells in *Populus tomentosa* were analyzed in our previous study (Yin et al., 2022). The results are summarized in **Table 1**.

**Table 1:** Results about the timing of differentiation and death of ray parenchyma cells in *Populus tomentosa*.

	Radial position in xylem	Number of growth ring	Starch grain		Cell nuclei		Secondary wall deposition		Lignification	
			Contact cell	Isolation cell	Contact cell	Isolation cell	Contact cell	Isolation cell	Contact cell	Isolation cell
*R1*	The current-year xylem	1^st^	+	+++	+++	+++	+	–	+	–
*R2*	Middle sapwood	15^th^	+	++	+++	+++	+++	–	+++	–
*R3*	Inner sapwood	20^th^	+	+	++	+++	+++	–	+++	–
*R4*	Transition zone	23^rd^	–	+	+	++	+++	+	+++	+
*R5*	Middle heartwood	33^rd^	–	–	–	–	+++	+++	+++	+++

1) The changes in protoplasmic characteristics in ray parenchyma cells during the transformation from sapwood to heartwood were from our previous study (Yin et al., 2022). The presence of starch grains in contact cells was revised by TEM observations.

2) +++ = abundant distribution, ++ = moderate distribution, + = infrequent distribution, - = no distribution.

### Wood maceration

Wood maceration (Franklin, 1945) was used to obtain the aforementioned parameters. The protocol was as follows: wood samples were washed thoroughly with the 0.05 wt% phosphate buffer and trimmed into toothpick-size sticks. The sticks were digested in a 5:4:21 mixed solution of glacial acetic acid, hydrogen peroxide (30%), and deionized water at 80°C for 48 h. They were subsequently rinsed in distilled water and stained with safranine.

### Quantitative anatomic analysis of ray parenchyma cells

The quantitative variables of ray parenchyma cells, including cell length, cell width, and cell length/width ratio, were measured at *R1*~*R5*. After wood maceration, the images taken from the microscope (DP71, Olympus, Japan) were analyzed by ImageJ software (http://imagej.nih.gov/ij). The number of isolation cells used for quantitative analysis was 30 individuals for each radial position. Because contact cells were much more difficult to separate intact after maceration, the number of contact cells used for quantitative analysis was in the range of 7~29 individuals for *R1*-*R5*.

### Transmission electron microscope

Small blocks (T×R×L, approximately 2×1×1 mm^3^) of wood samples at *R1*~*R5* were washed with 0.05 wt% phosphate buffer and immediately put into 1 wt% OsO_4_ aqueous solution overnight. After washing four times with 0.05 wt% phosphate buffer, the blocks were dehydrated in a graded ethanol series (30 v/v%, 50 v/v%, 60 v/v%, 70 v/v%, 80 v/v%, 90 v/v%, 100 v/v%, 100 v/v%, and 100 v/v%). Following this, they were exchanged in the acetone:ethanol series (1:2, 1:1, 2:1) and then fully submerged in 100% acetone for 20 minutes. Finally, they were exchanged in a graded Spurr’s resin (ERL 4221: D.E.R 736:NSA: DMAE=10.0:6.5:26.0:0.4):acetone series (1:2, 1:1, 2:1) and polymerized in 100% Spurr’s resin at 40°C for 2 hours and then at 70°C for 48 hours. Ultrathin sections with a thickness of 100 nm were cut using a diamond knife on a microtome (EM UC7, Leica, Germany). The ultrathin sections were stained with 2% aqueous uranyl acetate, and examined under a transmission electron microscope (HT7700, Hitachi, Japan).

### Histochemical methods

Wood samples stored in the FAA solution were washed with water thoroughly and used to prepare radial wood slices with a thickness of 20 μm by sliding microtome (SM, 2010R, Leica, Germany). The presence of phenolic compounds was determined using Fast Blue B salt (O’Brian and McCully, 1981) to give a characteristic reddish-brown reaction product. The vanillin-HCl test was also applied to distinguish condensed tannins (Gardner, 1975). The images were taken from the microscope (DP71, Olympus, Japan).

### Statistical analysis

Two-way analyses of variance (ANOVAs) were conducted to investigate the effects of radial position and ray cell type on the cellular traits of ray parenchyma cells. Each set of grouped data was analyzed separately, employing Equation 1:


(1) $$Y_{ijk}=\mu +\alpha_{i}+\beta_{j}+{\left(\alpha \beta \right)}_{ij}+\epsilon_{ijk}$$

where *Y_ijk_* is the response variable for the *i*_th_ radial position, *j*_th_ ray cell type, and *k*_th_ observation. *μ* is the overall mean. *α_i_* represents the effect of the *i*_th_ radial position. *β_j_* represents the effect of the *j*_th_ ray cell type. (*αβ*)*_ij_* is the interaction effect between the *i*_th_ radial position and the *j*_th_ ray cell type. ϵ*_ijk_* is the random error component. A Box-Cox transformation was performed on the data prior to the ANOVA to satisfy the assumptions of normality and homogeneity of variances. The assumption of normality was evaluated using Shapiro’s test, while the assumption of homogeneity of variances was evaluated using Levene’s test. *Post-hoc* comparisons among the means were made using Tukey’s HSD test to identify specific differences between the levels of factors. Differences were considered to be significant at a P-value of less than 0.05 in the ANOVA F-test. All statistical tests were performed using R version 4.3.0 (R Core Team, 2023).

## Results

### Morphological analysis of two types of ray parenchyma cells during the transformation from sapwood to heartwood

**Figure 1:**
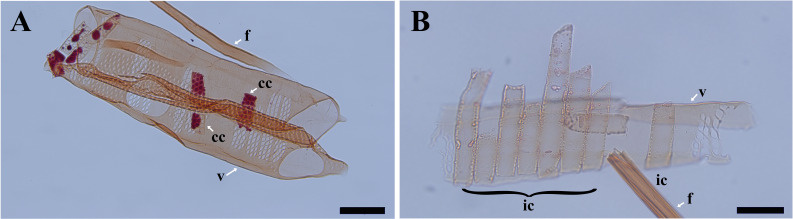
Light micrographs of macerated cells in *Populus tomentosa*. **(A)** the fiber, vessel elements, and contact cells at *R5*. **(B)** the fiber, the vessel element, and isolation cells at *R1*. cc, contact cell; ic, isolation cell; f, fiber; v, vessel element. Scale bars: A 100 μm, B 50 μm.

After wood maceration, it is vividly shown that ray parenchyma cells in contact with vessels have characteristic vessel-ray pits (**Figure 1A**) and are thus considered to be contact cells. Ray parenchyma cells in the center of rays are isolated from pit-mediated connections with vessels (**Figure 1B**), thus, they are isolation cells.

**Figure 2:**
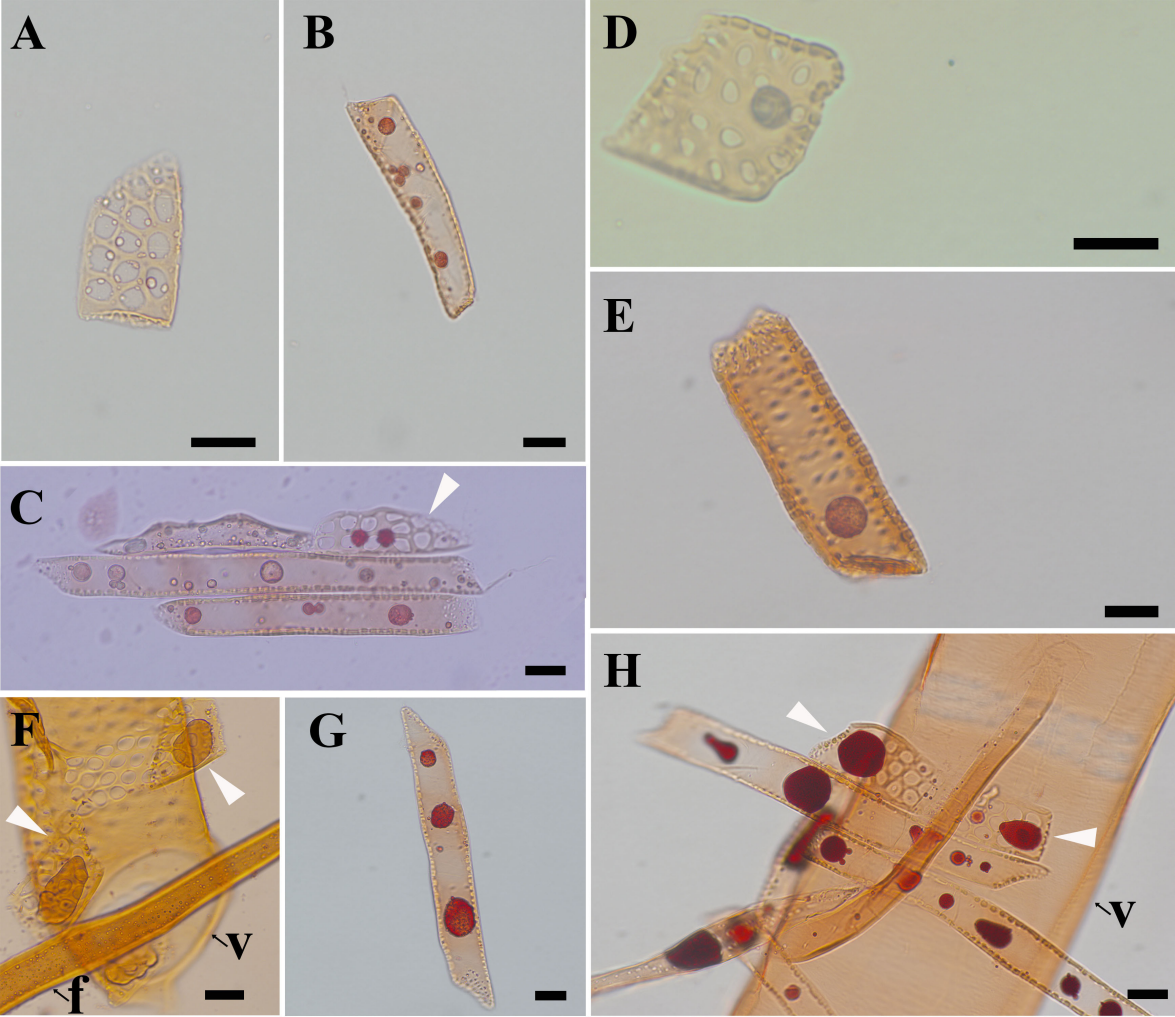
Light micrographs of macerated xylem cells, stained with safranine, showing droplets in ray parenchyma cells. **(A)** contact cells at *R1*, **(B)** isolation cells at *R1*, **(C)** ray parenchyma cells at *R2*, **(D)** contact cells at *R3*, **(E)** isolation cells at *R3*, **(F)** contact cells at *R4*, **(G)** isolation cells at *R4*, and **(H)** ray parenchyma cells at *R5*. f: fiber, v: vessel element. White arrows in C, F, and H indicate contact cells. Scale bars: A-D, F-H 20 μm, E 50 μm.

**Figure 3:**
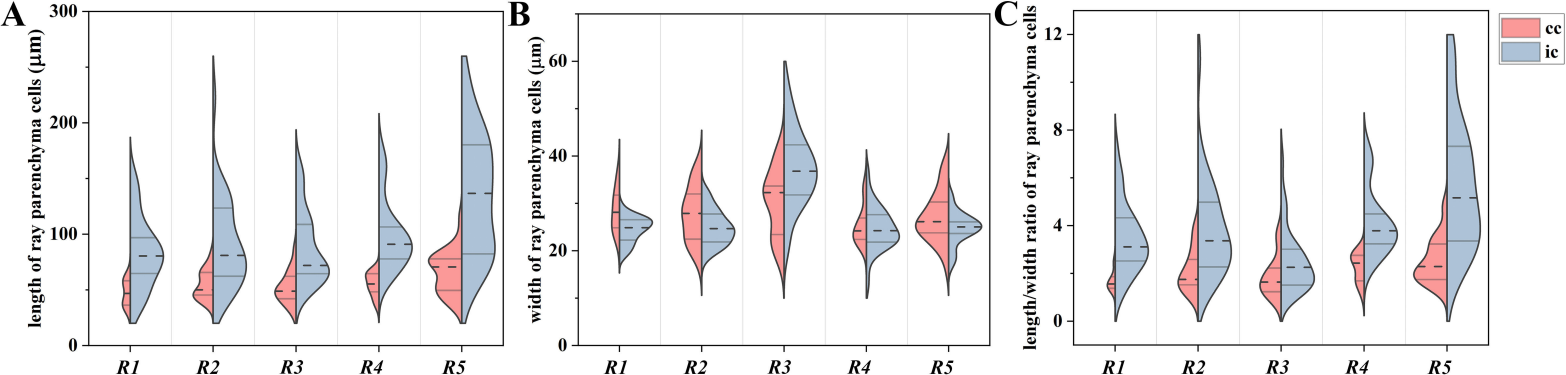
Split-violin plots exhibiting cell size distributions of ray parenchyma cells at *R1*~*R5* of *Populus tomentosa*. **(A)** cell length, **(B)** cell width, and **(C)** cell length/width ratio. Brick red and light blue denote contact cells (cc) and isolation cells (ic), respectively. The top and bottom of the lines within the split violin depict the 75^th^ and 25^th^ percentiles of the distribution. The dashed line within the split violin signifies the median values.

The morphological changes of contact cells were compared with those of isolation cells during the transformation from sapwood to heartwood (**Figure 2**). The most obvious changes were the accumulation of droplets and the shape and size of ray parenchyma cells. Pale-colored droplet substances were first seen in ray parenchyma cells at *R1*, as shown in *Populus sieboldii*×*P. grandidentata* (Nakaba et al., 2012). Droplets increased remarkably in size during the transformation from sapwood to heartwood (Kampe and Magel, 2013). Dark-colored droplet substances were evident in both contact cells and isolation cells at *R5* (**Figure 2H**). Furthermore, quantitative analysis showed profound changes in morphological traits. Aided by a two-factor ANOVA analysis, it was revealed that cell length, cell width, and cell length/width ratio were all significantly affected by both radial position and ray cell type (P<0.01). Split violin plots show visual comparisons of cell size between contact cells and isolation cells and are demonstrated in **Figure 3**.

Isolation cells presented a more elongated shape than contact cells, but no significant difference was shown between contact cells and isolation cells in terms of cell width. The length of contact cells increased radially with mean values from 47.2 μm to 65.9 μm during the transformation from sapwood to heartwood. Isolation cells also elongated from an average of 82.5 μm at *R1* to 135.0 μm at *R5*. Regarding the cell width, contact cells had a minimal value of 25.3 μm at *R4* and a maximum of 30.3 μm at *R3*. The width of isolation cells reached a maximum of 37.5 μm at *R3* but varied slightly among other radial positions. Consequently, the cell length/width ratio of contact cells reached a maximum of 2.5 at *R5* and a minimum of 1.7 at *R1*, and that of isolation cells had a maximum of 5.7 at *R5* and a minimum of 2.5 at *R3*. The results demonstrated that the cell length/width ratio of ray parenchyma cells reached a maximum in heartwood.

Isolation cells were significantly longer than contact cells (P<0.01), but no significant difference was shown between contact cells and isolation cells in terms of cell width (P=0.235). Therefore, isolation cells had a larger cell length/width ratio than contact cells (P<0.01) in all five radial positions, presenting a more elongated shape than contact cells (**Figure 2**). The cell length/width ratio of isolation cells was almost two times larger than that of contact cells for each xylem radial position (**Figure 3**). Moreover, differences in cell sizes among *R1*~*R5* were more obvious in isolation cells when compared with contact cells. Meanwhile, cell size variability was more marked in isolation cells, especially in the case of cell length (**Figure 3A**).

### Ultrastructural analysis of two types of ray parenchyma cells during the transformation from sapwood to heartwood

**Figure 4:**
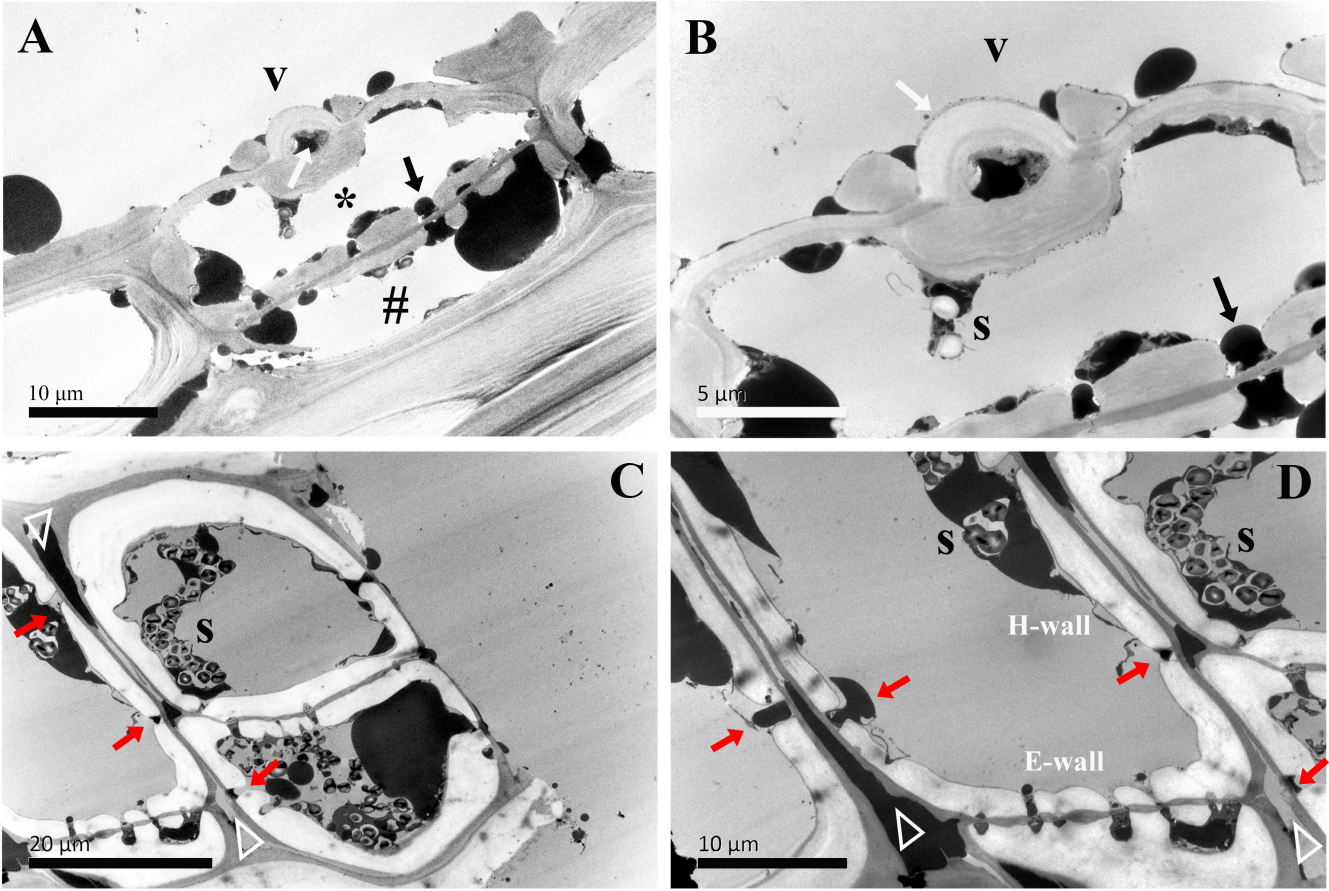
TEM micrographs of radial sections of ray parenchyma cells at *R1*, the current-year xylem, showing starch grains and dark substances in the cell content. The planes of sections are slightly oblique. **(A)** the contact cell (asterisk, *) and the connection of the isolation cell (#) with ray pits, **(B)** the contact cell with a vessel-ray pit (white arrow). Dark droplet substances are transported between the contact cell and the adjacent isolation cell through ray pits. **(C, D)** contact cells. Blind pits (red arrows) face the intercellular spaces (white triangles), and dark droplet substances are released from isolation cells into the intercellular spaces through the blind pits. The intercellular space leading in a longitudinal direction through the pit membrane is filled with dark substances. V: vessel element, H-wall: horizontal wall of the ray parenchyma cell, E-wall: end wall of the ray parenchyma cell, and S: starch grains.

**Figure 5:**
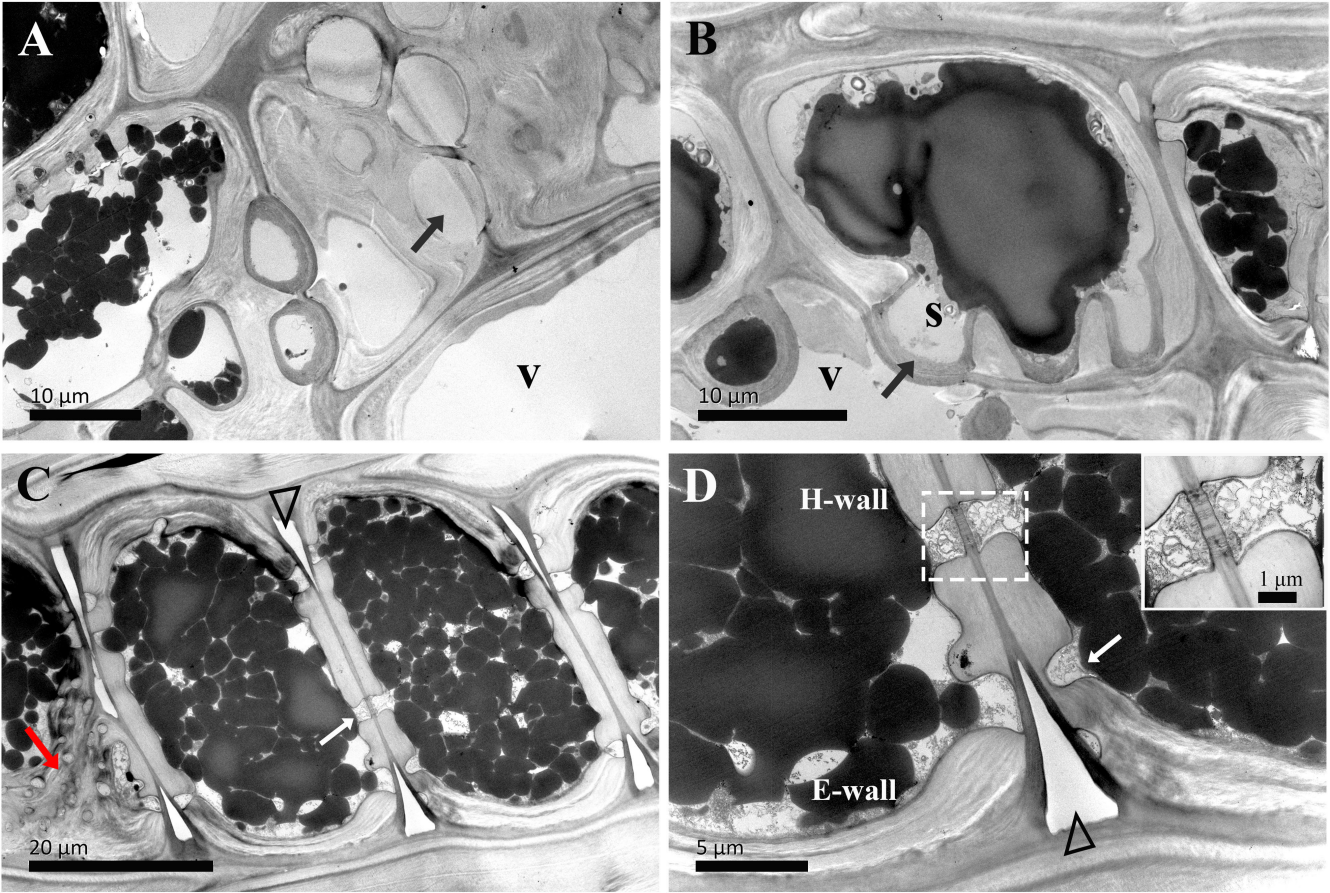
TEM micrographs of radial sections of ray parenchyma cells at *R2*, the middle sapwood, showing starch grains and dark substances in the cell content. The planes of sections are slightly oblique. **(A, B)** Contact cells with vessel-ray pits (black arrows). **(C, D)** Isolation cells and the connection with ray pits (white arrows) on the horizontal walls. Sieve-like pits on the end walls are marked by red arrows **(C)**. A close-up of radial translocation in rays is inset in the top right in D, note the prominent pit canal, the plasmodesmata are clearly evident, and dark substances are transported between the two adjacent isolation cells through the plasmodesmata. The triangle indicates the intercellular space. V: vessel element, H-wall: horizontal wall of the ray parenchyma cell, E-wall: end wall of the ray parenchyma cell, and S: starch grains.

**Figure 6:**
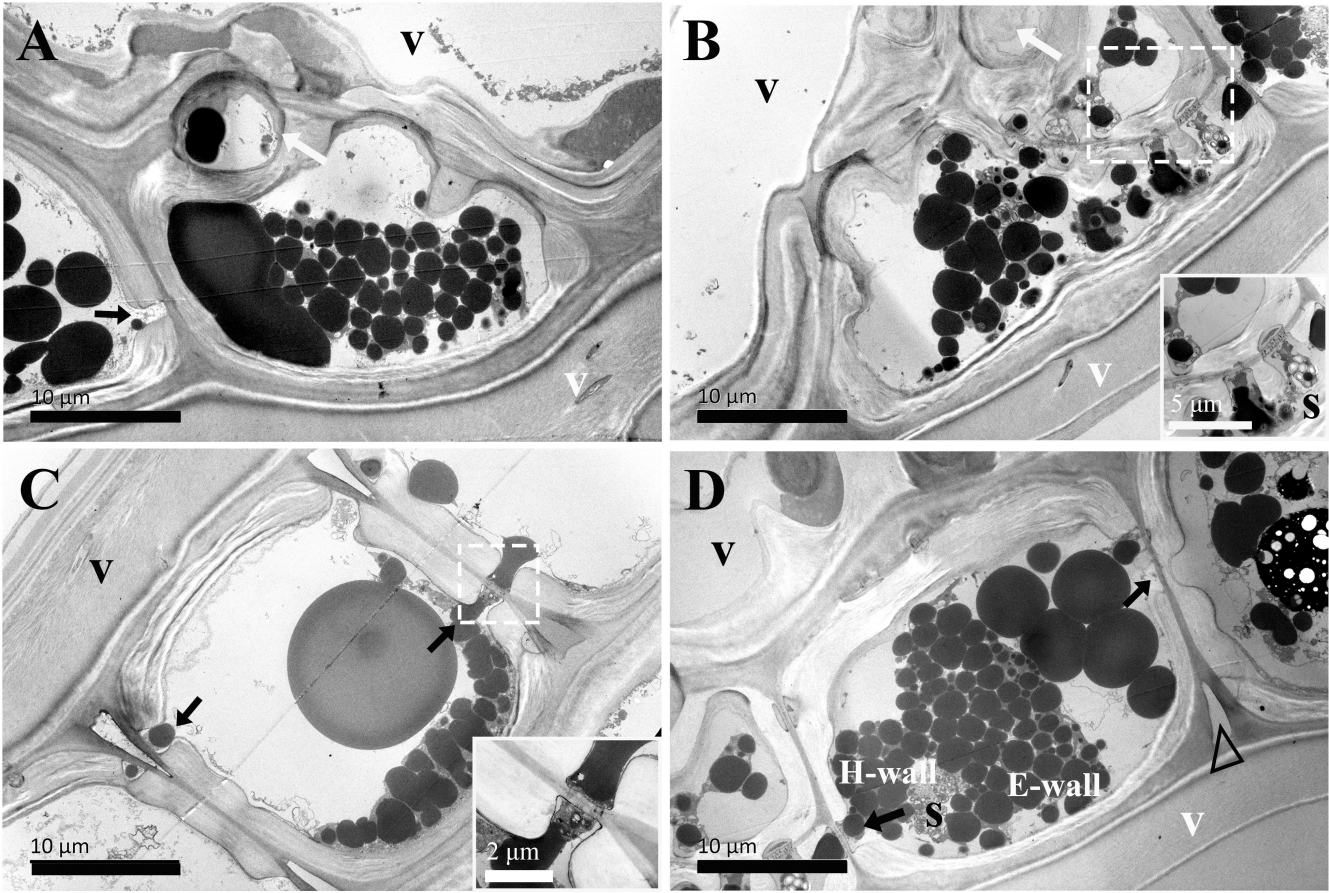
TEM micrographs of radial ultrathin sections of ray parenchyma cells at *R3*, the inner sapwood, showing starch grains and dark substrates in the cell content. The planes of sections are slightly oblique. **(A, B)** contact cells with vessel-ray pits (white arrows) and ray pits (black arrows). Plasmodesmata is highly magnified and inset in the bottom right in B, dark substances are transported between the two adjacent contact cells through plasmodesmata. **(C, D)** isolation cells connected with ray pits (black arrows) on the horizontal walls. Plasmodesmata is highly magnified and inset in the bottom right in **(C)** Dark substances are transported between the two adjacent isolation cells through plasmodesmata in ray pits. The triangle indicates the intercellular space. V: vessel element, H-wall: horizontal wall of the ray parenchyma cell, E-wall: end wall of the ray parenchyma cell, and S: starch grains.

**Figure 7:**
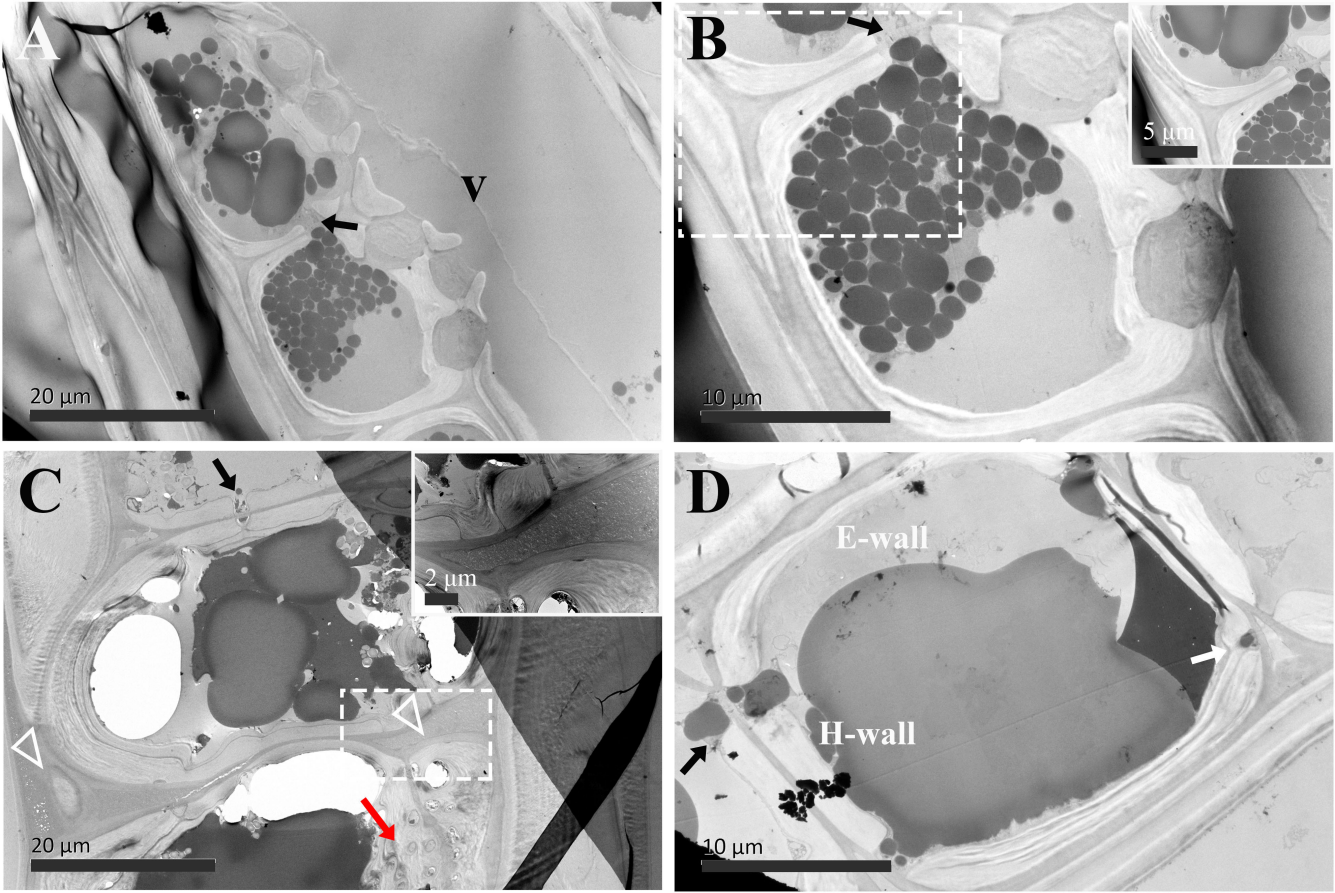
TEM micrographs of radial sections of ray parenchyma cells at *R4*, the transition zone, showing dark substrates in the cell content. The planes of sections are slightly oblique. **(A, B)** Contact cells, the cell corner is highly magnified and inset in the top right in **(B)** Dark substances are transported between the two adjacent contact cells through ray pits (black arrow). **(C, D)** Isolation cells connected with ray pits (black arrows) on the horizontal walls. The triangle indicates the intercellular space, which is highly magnified and inset in the top right in **(C)** Sieve-like pits on the end walls are marked by a red arrow. Dark droplet substances are released from isolation cells into the intercellular spaces through the blind pit (white arrow). V: vessel element, H-wall: horizontal wall of the ray parenchyma cell, and E-wall: end wall of the ray parenchyma cell.

**Figure 8:**
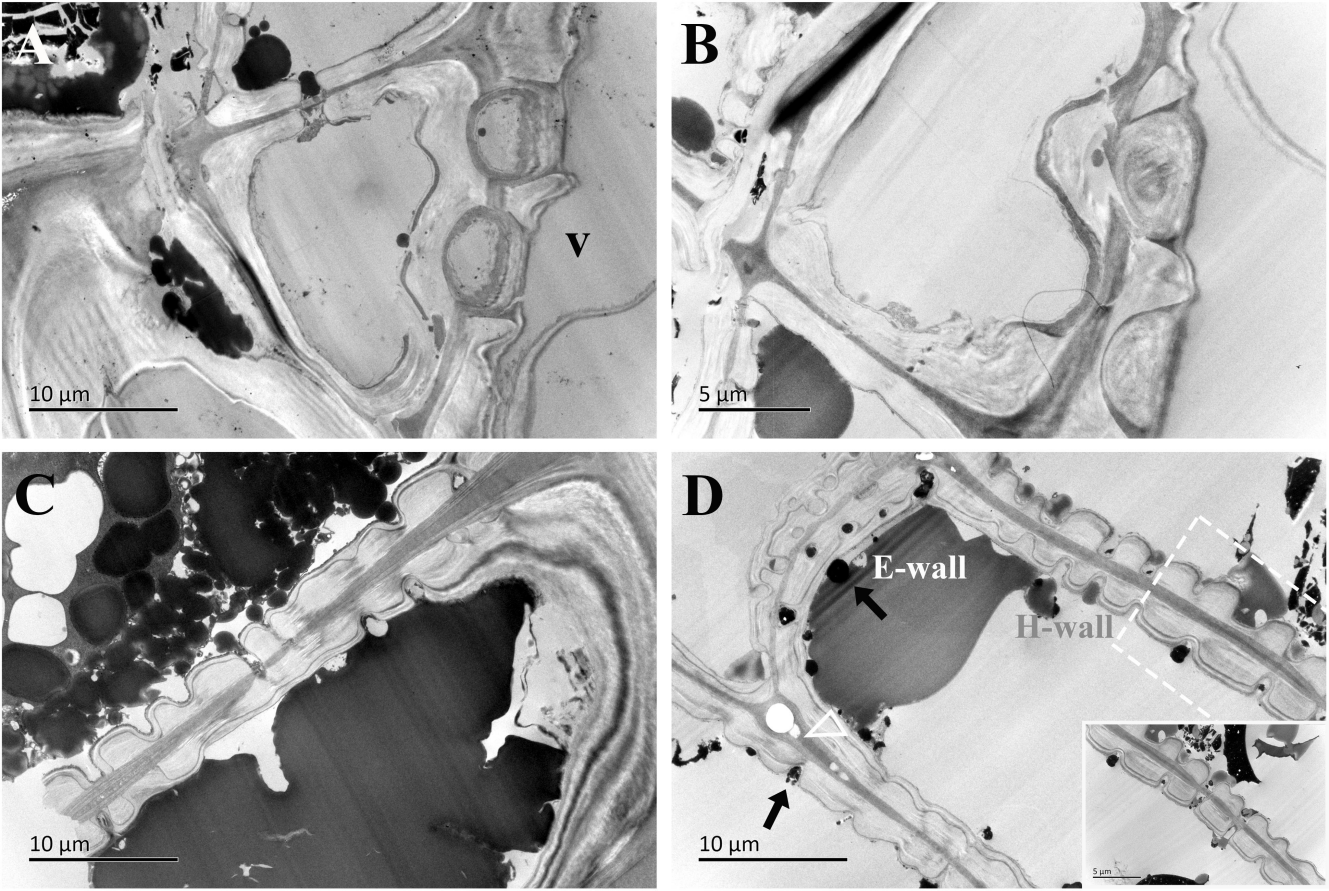
TEM micrographs of radial ultrathin sections of ray parenchyma cells at *R5*, the middle heartwood, showing dark substrates in the cell content. The planes of sections are slightly oblique. **(A, B)** Contact cells with vessel-ray pits. **(C, D)** Isolation cells connected with ray pits (black arrows). The horizontal wall of the isolation cell is highly magnified and inset in the bottom right in **(D)** Dark substances are transported between the two adjacent isolation cells through ray pits. The triangle indicates the intercellular space. V: vessel element, H-wall: horizontal wall of the ray parenchyma cell, and E-wall: end wall of the ray parenchyma cell.

The ultrastructural changes of two types of ray parenchyma cells underlying the transformation from sapwood to heartwood were elucidated (**Figures 4**–**8**). The vessel-ray pit is characteristic of contact cells on the radial ultrathin sectional views, as marked by white arrows in **Figures 4A, B**.

In the current-year xylem (*R1*), secondary cell wall deposition had already begun in contact cells, but not yet started in isolation cells (Yin et al., 2022). Isolation cells had a thick primary wall in the current-year xylem (**Figures 4C, D**). As shown in **Figure 4B**, the cell wall in contact cells showed a typical polylamellate structure, and boundaries among cell wall layers could not be exactly delineated. A closer inspection of the isolation cell walls, as shown in **Figure 4D**, revealed a dark layer lying inside the primary wall. Likewise, the isolation cells of *Populus tremuloides* within a single growth season were also coated inside with a dark layer, called an isotropic layer (Chafe and Chauret, 1974). Furthermore, it can be clearly seen that there are many blind pits in the isolation cells (**Figures 4C, D**). Intercellular spaces are pathways for gas exchange, conduits for water and nutrient transport or storage, and storage sites and diffusion pathways for heartwood substances (Nagai and Utsumi, 2012). These blind pits face the intercellular spaces, and intensely stained dark substances are released from isolation cells into the intercellular spaces through the blind pits.

The ultrastructural features of ray parenchyma cells in the region of middle sapwood (*R2*) are depicted in **Figure 5**. Contact cells formed an additional layer on the inner side of the secondary wall in middle sapwood (**Figures 5A, B**). This was similar to those already reported, i.e., the amorphous layer within the contact cells of *Populus maximowiczii* (Murakami et al., 1999). This layer was previously called the protective layer. While the ultrastructure of isolation cells was not significantly altered in the region of middle sapwood, an isotropic layer on the inner side of the primary wall was characteristic (**Figures 5C, D**). This is in accordance with ray parenchyma cells in the sapwood of *Pinus lambertiana* Dougl., which were located at 10 rings from the cambium (Bamber, 1976). The intercellular spaces between isolation cells were still wide.

In the inner sapwood (*R3*), the ultrastructure of both contact cells and isolation cells was not altered. A polylamellate structure remained a characteristic of contact cells and had a protective layer on the inner side of the secondary wall (**Figures 6A, B**). The isotropic layer on the inner side of the primary wall was still a characteristic of isolation cells (**Figures 6C, D**). They obviously differed in the ultrastructural feature from the neighboring vessel element (**Figure 6B**). Wide intercellular spaces were still apparent between isolation cells.

In the transition zone (*R4*), most contact cells died, in contrast, isolation cells still lived (**Table 1**). There was no ultrastructural change in contact cells (**Figures 7A, B**). They were still in the distinguishing polylamellate structure. On the contrary, it is worth noting that secondary thickening and lignification started in isolation cells in the transition zone (**Table 1**). As shown in **Figures 7C, D**, a thin secondary wall was deposited on the thick primary wall. There was also no evidence of clear interlayer boundaries in the secondary wall of isolation cells. Moreover, an additional isotropic layer coated inside was noted (**Figure 7C**). Our findings related to the two isotropic layers are mostly in accordance with isolation cells in the outer two annual rings of 3-year-old branches of 6- to 9-year-old poplar trees (*Populus* × *canadensis* Moench ‘*robusta*’) (Sauter and van Cleve, 1990). Isolation cell walls are in a polylamellate structure, without evidence of clear interlayer boundaries (**Figures 7C, D**). Furthermore, it is remarkable that a significant change occurred in the intercellular space. A higher magnification of the intercellular space of isolation cells is shown in **Figure 7C**. Material depositions in the intercellular space became apparent and the intercellular layer became denser.

In the region of middle heartwood (*R5*), contact cells were still in the polylamellate structure, while the protective layer of contact cells was more pronounced than that in the transition zone (**Figures 8A, B**). Isolation cells had fully died in this region. Two isotropic layers became much more evident. Moreover, high electronic adsorptions were found in intercellular spaces.

**Figure 9:**
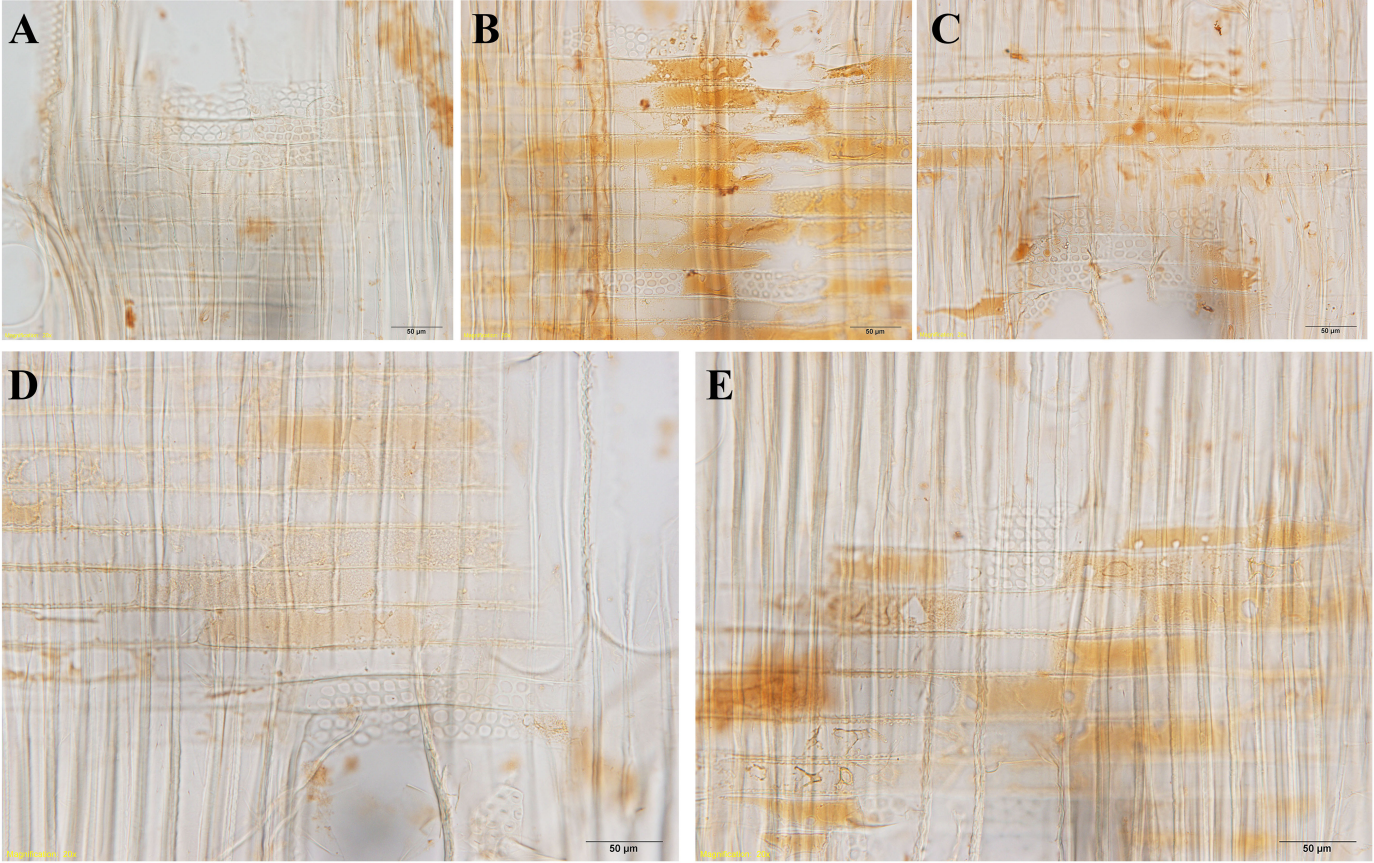
Light micrographs of radial wood sections, stained with Fast Blue B salt, showing droplets in ray parenchyma cells at *R1*~*R5* in xylem parts. **(A)** *R1*, **(B)** *R2*, **(C)** *R3*, **(D)** *R4*, and **(E)** *R5*. Scale bars: 50 μm.

**Figure 10:**
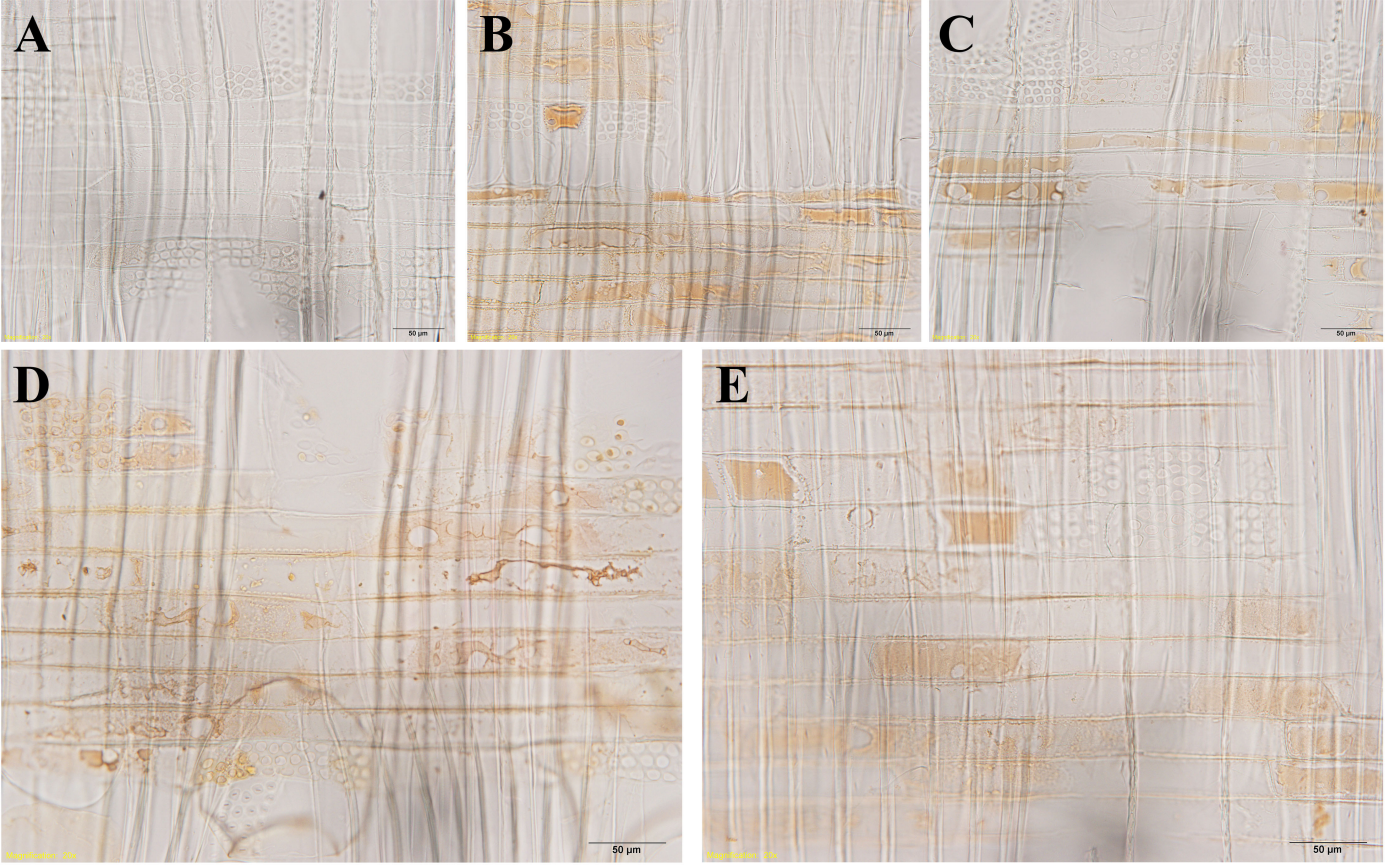
Light micrographs of radial wood sections after the vanillin-HCl test, showing droplets in ray parenchyma cells at *R1*~*R5* in xylem parts. **(A)** *R1*, **(B)** *R2*, **(C)** *R3*, **(D)** *R4*, and **(E)** *R5*. Scale bars: 50 μm.

The senescence of ray parenchyma cells was accompanied by the accumulation of extractives or colored substances, as reported in *Taiwania cryptomerioides* (Chen et al., 2018). Dark lipid droplets were evident in both contact cells and isolation cells at *R1*~*R5*. Using ultra-thin sections stained by osmium tetroxide, dark lipid droplets with high electron absorption were assumed to be organic compounds containing double bandings or alcoholic OH-groups that react with OsO_4_, i.e., resinous and polyphenolic compounds (Fengel, 1970). Furthermore, the positive reactions of phenolics with Fast Blue B salt (**Figure 9**) and tannins with vanillin-HCl (**Figure 10**) proved that these dark-colored lipid droplets were polyphenols.

The passage of starch grains and polyphenolic droplets was also observed macroscopically in this work. Ray pits are involved in intercellular diffusion pathways, which can be easily visible on horizontal walls and end walls in a sectional view. Ray parenchyma cells lived throughout the sapwood. Living ray cells could connect to each other via numerous strands of plasmodesmata in ray pits, which function in the symplastic movement of metabolites. Polyphenols seemed to migrate from one ray parenchyma cell to the adjacent one by passing the plasmodesmata in ray pits. This process was represented in ray pits between two adjacent isolation cells (inset in the top right in **Figure 5D**) and in ray pits between two adjacent contact cells (inset in the bottom right in **Figure 6B**). Furthermore, it was quite obvious that sieve-like pits on the end walls of isolation cells, as depicted by the red arrow in **Figure 5C**, differed with respect to size and density compared with pits on the horizontal wall. These features facilitate radial transport from one isolation cell to the next. The intercellular spaces of isolation cells also served as pathways for extracellular diffusion of heartwood substances. Polyphenols were coming out of ray blind pits in isolation cells, transported, and accumulated in the intercellular space (**Figures 4C**, **6D**). Polyphenolic constituents reached the intercellular spaces through blind pits, and it was concluded the spaces serve as a deposit of the constituents.

## Discussion

The cell walls of both contact cells and isolation cells are distinct from the wood fiber cell wall, which is characterized by a thin lignified primary wall and a thick secondary wall with the latter composed structurally of three characteristic layers (S_1_, S_2_, S_3_) (Fernando et al., 2023). Moreover, the positional difference of ray parenchyma cells was an important factor in the ultrastructural variation during the transformation from sapwood to heartwood. The most conspicuous feature of the ultrastructure was the initiation of secondary wall thickenings and lignification occurring earlier in contact cells in the current-year xylem, however, isolation cells formed thick primary walls coated inside with a dark layer, called an isotropic layer, at the end of the growing season but did not lignify, nor form secondary walls until the transition zone. Resulting from the influence of short-lived vessel elements, the secondary wall thickening of contact cells developed earlier than that of isolation cells, as found in *Populus maximowiczii* (Murakami et al., 1999) and *Populus sieboldii* × *P. grandidentata* (Nakaba et al., 2012). The secondary wall thickening and lignification of cell walls could provide biomechanical support and defense against fungal pathogens for contact cells in sapwood. The delayed formation of the secondary walls of isolation cells could possibly facilitate the readjustment of the position of the cells in the differentiating xylem that is a consequence of differences in the extent of expansion (Kitin et al., 2003) and be beneficial for wall stress relaxation during growth (Cosgrove, 1997). Furthermore, a very abrupt change of ultrastructure exists in the transition zone. In this region, secondary thickening and lignification started in isolation cells. Meanwhile, the secondary wall in isolation cells was much thinner, an additional isotropic layer was formed on the inner side of the secondary wall of isolation cells. Instead, the ultrastructure of contact cells was not altered during the transformation from sapwood to heartwood. A protective layer on the inner side of the polylamellate-structured secondary wall was characteristic of contact cells. Therefore, in the region of sapwood, lignified secondary cell walls for contact cells and thickened primary cell walls for isolation cells clarified that positional differences of ray parenchyma cells influenced the ultrastructural variation during the transformation from sapwood to heartwood.

Radial transport beyond growth rings is the major task of the xylem ray parenchyma cell. Parenchyma cell size and shape are prime indicators of function (Carlquist, 2018). Storage cells in rays can be distinguished from flow cells by size and shape, by fewer and smaller pits, and by contents (Carlquist, 2018). Ray parenchyma cells elongated towards the heartwood. Moreover, isolation cells had a greater cell length/width ratio, and sieve-like pits were only present on the end walls in isolation cells. The present observations suggest that isolation cells could be primarily designed for radial transport, in comparison with contact cells. These further indicated that isolation cells provided a more favorable channel for material transport during the transformation from sapwood to heartwood.

Heartwood substances are generally synthesized in living ray parenchyma cells at the transition zone, released into the intercellular spaces, and then diffused into the neighboring heartwood tissues (Zhang et al., 2009). Storage of carbohydrates in the living cells plays a crucial role in the synthesis of heartwood constituents (Magel et al., 1994). Starch grains, used for the energetic biosynthesis of secondary metabolites and as structural feedstocks, were abundant in isolation cells compared with contact cells. A significant decrease in terms of the number of starch grains became evident in the transition zone. Starch depletion during the transformation from sapwood to heartwood and the accumulation of large amounts of polyphenolic droplets probably indicated that storage material, mainly starch, was consumed or withdrawn to provide the carbon sources for the biosynthesis of heartwood substances in the heartwood formation process (Chen et al., 2018). Meanwhile, the dark droplets in isolation cells lost their spherical shape and flowed together, in accordance with the amounts of osmiophilic aggregates in ray parenchyma cells in the heartwood of *Crytomeria japonica* (Arakawa et al., 2018). In addition, material deposition in the intercellular spaces became pronounced in the transition zone, probably resulting from the oxidation of heartwood substances triggered by the gas from the intercellular spaces (Nagai and Utsumi, 2012). These observations suggested that ray parenchyma cells of different radial positions in the xylem part might act independently and contribute to the synthesis of heartwood substances. Contact cells that die earlier are not involved in the formation of heartwood extractives. Isolation cells actively synthesize heartwood substances prior to their death. Similar observations were also previously reported in *Cryptomeria japonica* (Nobuchi et al., 1982; Nobuchi and Harada, 1985) and in *Pinus densiflora* and *Pinus rigida* (Nakaba et al., 2008).

The present observations suggest that positional information appears to be an important factor in the transformation from sapwood to heartwood. Contact cells and isolation cells might have different functions in heartwood formation. Ray parenchyma cells in contact with vessel elements do not play a role in heartwood formation. By contrast, isolation cells were flow cells, specializing in radial transport, and were involved in the synthesis of heartwood substances.

## Conclusion

The structural changes that occur in parenchyma cells within a ray and their proximity to neighboring short-lived vessel elements during the transformation from sapwood to heartwood give some indications of the complicated system within xylem rays involved in heartwood formation. Isolation cells presented a more elongated shape than contact cells. The secondary wall thickening and lignification of contact cells developed in the current-year xylem, much earlier than isolation cells. On the contrary, in sapwood, isolation cells had a characteristic isotropic layer on the inner side of the thick primary wall. A very abrupt variation in ultrastructure existed in isolation cells in the transition zone. Secondary thickening and lignification started; a thin secondary wall was deposited on the thick primary wall of isolation cells, and an additional isotropic layer was coated inside. Nevertheless, most contact cells died in the transition zone and no alteration in the ultrastructure was observed. Taken as a whole, these results emphasize that ray parenchyma cells in contact with vessels do not play a role in the synthesis of heartwood substances. By contrast, isolation ray parenchyma cells are flow cells, specializing in radial transport, and are involved in the synthesis of heartwood substances.

## Data Availability

The raw data supporting the conclusions of this article will be made available by the authors, without undue reservation.
